# Solution-focused approaches to improving the careers of women academics in psychiatry: UK viewpoint

**DOI:** 10.1192/bjo.2025.10845

**Published:** 2025-09-08

**Authors:** Angela Hassiotis, Lindsey Sinclair, Anne Lingford Hughes, Allan H. Young, Ania Korszun, Emmeline Lagunes-Cordoba, Kenneth R. Kaufman

**Affiliations:** University College London, UK; and North London Foundation NHS Trust, London, UK; University of Bristol, UK; Imperial College London, UK; Queen Mary University of London, UK; North London Foundation NHS Trust, London, UK; Rutgers Robert Wood Johnson Medical School, USA

**Keywords:** Education and training, careers, clinical academic survey, equality diversity inclusion (EDI)

## Abstract

Clinical academics in psychiatry face several inequities, many of which are specific to women academics and intersectional in nature. We characterise the current state of UK academic psychiatry utilising findings of the annual Medical Schools Council clinical academic survey, and consider initiatives seeking to address gaps in supporting the career journeys of women academics.

**Figure:**
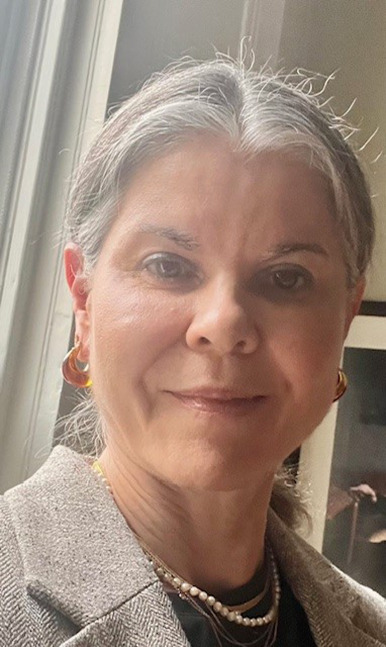

A significant body of evidence has accumulated^1–3^ suggesting persistent structural inequalities that impact the career progressions of women academics within medicine. Inequalities can occur across the career pipeline, from the earliest training stages to senior positions, and notably include lack of diversity, exclusive environments, fewer opportunities for mid/senior-level appointments (e.g. senior lecturer and reader), intensely competitive research funding, working patterns that may adversely affect those with caring responsibilities, lack of role models/mentorship and unequal pay. A recent qualitative study examining the impact of the COVID-19 pandemic^4^ on clinical academics found that gendered roles (e.g. caring responsibilities) disproportionately affected the academic productivity of women academics, and those from minority ethnic groups, who experienced additional challenges and were potentially motivated to leave academia.

The findings of the annual Medical Schools Council (MSC) clinical academic survey (https://www.medschools.ac.uk/clinical-academic-survey) in the UK raise some important points about the academic pathway across all medical specialities, and in psychiatry specifically. The annual survey may have had some impact, in addition to other initiatives including the launch of the National Institute of Health and Care Research (NIHR) in 2006, on the increase in full-time equivalent (FTE) posts by 2010.

However, the survey data (2004–2023) indicate that there has been a reduction in all full-time and less-than-full-time clinical academic posts in psychiatry (lecturer, senior lecturer/reader, professor) of 26.2%, and a decline in National Health Service (NHS) funding for early careers. During the same period, there appears to have been an increase or no change in posts occupied by women at professorial and lecturer grades, respectively, and the loss of almost half of the posts at the associate professor (senior lecturer/reader) grade. We note that, despite the improved female to male ratios, there is substantial reduction in the numbers of women at associate professor grade which, combined with more women clinical academics in the 36- to 45-year age range being at lecturer level, suggests a pipeline problem. Loss of male posts is also of note in the same period, and may allude to other drivers outside the remit of this commentary (see Table 1).

**Table 1:** Total clinical academic posts in psychiatry 2004–2023, UK by gender^a^

Professors		Associate professors		Lecturers	
Men	Women	Men	Women	Men	Women
97 versus 86 (11.3% reduction)	20 versus 34 (70% increase)	108 versus 52 (51.9% reduction)	52 versus 28 (46.2% reduction)	25 versus 19 (24% reduction)	14 versus 14 (no change)
Female:male ratio, 20.6 versus 39.5% (increase)		Female:male ratio, 48.1 versus 53.8% (increase)		Female:male ratio, 56 versus 73.7% (increase)	
**Total posts**	316 versus 233 (decreased by 26.3%)				

a. Includes full-time equivalent (FTE) and less-than-FTE posts.

Geographically, Northern Ireland shows a recent increase in posts (senior, mid and early levels) but none occupied by women. All UK nations have experienced reductions in clinical academic positions; however, Wales has been disproportionately affected, partly due to its historically lower number of NHS-funded academic posts. Women working in regions with poor retention of clinical academics are therefore likely to encounter even greater challenges in pursuing a career in academic psychiatry. The vast majority of clinical academics are of White ethnicity, followed by Asian/British Asian and a significant minority not reported. Currently, the survey offers no intersectional data about the ethnicity of men and women clinical academics, but 2023 data show that there are five times more White clinical academics at professorial level, which reduces to three times as many at associate professor level, but it is an almost exclusively White background in early careers. Despite this being a more balanced picture than 15 years ago, lack of diversity at all levels remains an ongoing challenge that we have yet to overcome, and may impact women’s academic careers. This is more evident for speciality and associate specialist (SAS) doctors, whose roles are mainly focused on delivering clinical care and who find it hard to enter academic psychiatry in the UK.^5^ If we consider that most of the psychiatry SAS workforce are women, international medical graduates and from ethnic minorities, we can argue that there are exacerbating inequities confronted by this group of women professionals when trying to develop or maintain a career in academic psychiatry.

Finally, in the early stages of clinical academic careers when individuals take on opportunities to advance their career, more women are likely to work less than full time. This has been identified as a particular issue when seeking promotion or senior leadership appointments, because their productivity is often compared against those working full time. This is a cause of a systemic disadvantage that continues to limit women advancing to senior posts in which they could help address these issues. The existing literature confirms many of those trends as they are shared across academic medical specialities. Vassie et al^6^ identified 13 themes describing barriers to equitable participation in the clinical academic pathway, including personal, institutional/organisational factors and societal attitudes. They suggested that interventions to address any one individual barrier are unlikely to be successful and that multiple approaches are called for. Protected characteristics such as gender and race are likely to appear in disparity statistics; for example, although more women gain entry to medical schools, this does not translate into gender parity in academic careers, with a persistent gap especially at the most senior levels (available data relating to aggregated FTE and less than FTE indicate a 3.3:1 male:female professor ratio in 2023 for all academic specialties, and a 2.7:1 male:female professor ratio for psychiatry (source: MSC clinical academic survey).

The UK Department of Health and Social Care published a vision for the delivery of clinical research encompassing the creation of ‘a patient-centred, pro-innovation and digitally enabled clinical research environment’.^7^ A central part of research delivery is played by clinical academics. However, if the perception of insurmountable obstacles to a rewarding career in academia continues, the delivery of the long-term workforce plan will be compromised. It is recognised that the contribution of women clinical academics to psychiatric research is multifaceted and includes significant benefits for team collaboration and productivity, as well as a focused lens on research and clinical advancements into the mental health challenges of women patients.^8^ In not having sufficient women in senior posts, the voice of the new majority of UK doctors is not being adequately heard and is a disservice to the UK healthcare workforce.^9^ Furthermore, well-meaning initiatives such as the Scientific Women’s Academic Network Charter (Athena SWAN) for the advancement of gender equality in higher education have faced criticism, because adherence to the charter does not correspond to the pace of desired institutional change.^10^

Compared with other specialities, psychiatry (and mental health more broadly) is less able to rely on major charities such as the British Heart Foundation or Cancer Research UK, which support researchers with grants and personal career development awards. On top of this, Wellcome, a major funder of clinician scientists, appears to have shifted focus away from clinical academic trainees, potentially limiting the creation of more academic posts.^11^ The NIHR has, however, developed pathways to support a career in research in England through the NIHR Academy, coupled with initiatives to embed research culture within the NHS. The NHS continues to fund the majority of lecturer posts and a proportion of mid- and senior career posts, based on 2023 survey data.

Pinto da Costa et al^12^ clearly describe several barriers that must be overcome to ensure that women clinical academics have a clear path to a fulfilling career within a safe and inclusive environment. Specifically, the authors highlight gaps in mentorship availability, access to opportunities, systemic changes to support underrepresented groups and flexible working patterns. Although none of those points are necessarily new or specific to the UK, some changes have occurred that support women clinical academics; for example, research funders such as Wellcome and the Medical Research Council, have addressed potential financial loss relating to maternity leave and career breaks due to a range of caring duties. University psychiatry departments may achieve localised improvements by openly discussing promotion at all grades or launching funding opportunities for underrepresented and minoritised groups (UCL Division of Psychiatry EDI Chair, personal communication, 2024). Regular staff surveys, followed up by action plans and goals, can capture staff views on harassment and bullying, job satisfaction and workload, all of which impact women most often. Other proposals for increased investment in clinical academics include fair representation in both local Clinical Excellence Awards and National Clinical Impact Awards; however, the number of clinical academics holding such awards has fallen from 83 to 59% in the past 10 years. Organisations such as the Clinical Academic Training and Careers Hub (https://www.catch.ac.uk/), launched in 2021, while open to all clinical academics, can provide support and resources to academic psychiatrists on a range of topics e.g. equality diversity and inclusion groups/networks (see Supplementary Material Box 1 available at https://doi.org/10.1192/bjo.2025.10845 for a list of potential actions for various stakeholders).

A UK-wide complementary drive for recruitment to both academic psychiatry and psychiatry could be a useful addition to ongoing campaigns.^13^ Furthermore, the tutors who oversee trainees on the NIHR Integrated Academic Training Programme (*n* = 116, England) for psychiatry should examine the cohort composition to anticipate future trends and plan for retention and progression, because there were only seven doctoral fellowships available between 2019 and 2024. Employers, funders and other entities should strive to improve gender diversity and increase initiatives for minoritised groups to ensure their place in a future academic pipeline. Whatever approaches are considered, these need to be tailored to regional and national profiles, because we cannot have an increment of posts with none of them filled by women, as appears to have been happening in Northern Ireland. Such an endeavour must heed the suggestions made by Pinto da Costa et al^12^ in facilitating a sustainable progressive strategy for future career development of women clinical academics in psychiatry. Women clinical academics and allies in psychiatry need to keep on highlighting barriers, and pushing for changes to ensure that new generations of women psychiatrists can pursue a career in academia without those extra hurdles based on their gender.

## Supporting information

Hassiotis et al. supplementary material Hassiotis et al. supplementary material

## Data Availability

Data availability is not applicable to this article as no new data were created or analysed in this study.

## Supplementary material

The supplementary material is available online at https://doi.org/10.1192/bjo.2025.10845

## References

[1] Trusson D , Rowley E. Qualitative study exploring barriers and facilitators to progression for female medical clinical academics: interviews with female associate professors and professors. BMJ Open 2022; 12: e056364. 10.1136/bmjopen-2021-056364 PMC8921847 35288388

[2] Inguaggiato G , Pallise Perello C , Verdonk P , Schoonmade L , Andanda P , van den Hoven M , et al. The experience of women researchers during the Covid-19 pandemic: a scoping review. Res Ethics 2024; 20: 780–811.

[3] Araneda-Guirriman C , Sepúlveda-Páez G , Pedraja-Rejas L , San Martín J. Women in academia: an analysis through a scoping review. Front Educ 2023; 8: 1137866.

[4] Finn GM , Crampton P , Buchanan JA , Balogun AO , Tiffin PA , Morgan JE , et al. The impact of the COVID-19 pandemic on the research activity and working experience of clinical academics, with a focus on gender and ethnicity: a qualitative study in the UK. BMJ Open 2022; 12: e057655. 10.1136/bmjopen-2021-057655 PMC9184994 35676023

[5] Page M , Jackson D , Carty E. ‘I don’t belong anywhere’: identity and professional development in SAS doctors. Clin Med (Lond) 2024; 24: 100003. 38382180 10.1016/j.clinme.2023.100003 PMC11024821

[6] Vassie C , Smith S , Leedham-Green K. Factors impacting on retention, success and equitable participation in clinical academic careers: a scoping review and meta-thematic synthesis. BMJ Open 2020; 10: e033480. 10.1136/bmjopen-2019-033480 PMC7170560 32213518

[7] NHS England. *NHS Long Term Workforce Plan*, NHS England, 2023 (https://www.england.nhs.uk/wp-content/uploads/2023/06/nhs-long-term-workforce-plan-v1.21.pdf [cited 11 Dec 2024]).

[8] Bear JB , Woolley AW. The role of gender in team collaboration and performance. Interdiscipl Sci Rev 2011; 36: 146–53.

[9] General Medical Council. *More Female than Male Doctors for First Time Ever in the UK*. General Medical Council, 2025 (https://www.gmc-uk.org/news/news-archive/more-female-than-male-doctors-for-first-time-ever-in-the-uk#:~:text=Female%20doctors%20are%20greater%20in,with%20164%2C195%20men%20(49.96%25) [cited 9 Apr 2025]).

[10] Armstrong J , Sullivan A. A critical analysis of Athena Swan as a policy-scoring scheme. Br Educ Res J 2024; 51, 225–43

[11] O’Rahilly S. Academic clinician–scientists risk becoming an endangered species. Nat Med 2023; 29: 2989. 38087113 10.1038/s41591-023-02626-8

[12] Pinto da Costa M , Galderisi S , Herrman H , Riecher-Rössler A , Wasserman D. Breaking barriers in the professional development of women in academic psychiatry. BJPsych Open 2024; 10: e208. 39529275 10.1192/bjo.2024.808 PMC11698163

[13] Royal College of Psychiatrists. *Choose Psychiatry: Choose to Make a Difference*. RCPsych, n.d. (https://www.rcpsych.ac.uk/become-a-psychiatrist/choose-psychiatry [cited 28 Aug 2025]).

