# Supplementary material for: Solution-focused approaches to improving the careers of women academics in psychiatry: UK viewpoint
Source: BJPsych Open. 2025 Sep 8;11(5):e203. doi: 10.1192/bjo.2025.10845 (PMC12451557; doi:10.1192/bjo.2025.10845)
Supplement: Hassiotis et al. supplementary material [file S2056472425108454sup001.docx]

SUPPLEMENTARY MATERIAL

**Box 1. Solution focused approaches for supporting women clinical academics in Psychiatry in the UK**

| **Funders** | **Universities** | **NHS** | **RCPsych*** |
| --- | --- | --- | --- |
| Financial support during maternity leave or career breaks for caring responsibilities | Provision of financial support during maternity leave or career breaks for caring responsibilities | Joint job plans to align clinical and academic goals and pay progression | Support campaign to increase interest in academic psychiatry |
| Support early career academics by creating and maintaining career advancing opportunities, e.g. NIHR Academy | Discussion of promotion at appraisal meetings  Embedding family friendly policies across all departments | Support clinical academics to apply for local and national clinical impact awards | Make use of structures such as the Academic Faculty Executive and Associate Dean role for Academic Psychiatry to launch initiatives that will provide tailored responses to reversing decline in academic posts at national and local level |
| Promote initiatives in developing research leadership, e.g. NIHR Academy | Utilise findings from staff surveys and focus groups in order to address inequalities, e.g. on workload issues, harassment etc | Support academic aspirations of SAS** doctors e.g. at job plan meetings; promote mentorship opportunities and encourage research experience |  |
| Fund doctoral and post doctoral fellowships | Address pipeline issues for early and mid career clinical academics, e.g. develop networks (grades 8 and 9), run relevant seminars addressing the development of a research identity and future career goals | Through Research and Development support NIHR***initiatives of promoting research leadership in the NHS |  |
|  | Celebrate success and promote membership of organisations to enhance peer networks and access to resources and career advice | Celebrate academic success and promote membership of organisations to enhance peer networks and access to resources and career advice |  |
|  | Adopt and embed Athena Swan and relevant initiatives on intersectionality with a focus on women clinical academics |  |  |

*: Royal College of Psychiatry

**:Speciality and Associate Specialist (doctor)

***: National Institute for Health and Care Research
